# 

**Published:** 1890-04-19

**Authors:** 


**PRICE ONE PENNY.**

o. 186, Vol. VIII.—April 19th, 1890.

Registered at the General Post Office as a Newspaper for transmission in the United Kingdom and Abroad.

WITH EXTRA NURSING SUPPLEMENT.
Per Annum, 6s. 6d.; post free.
Monthly Parts, 6d.; post free 8d.
Ditto per Annum 8s. post free.

